# How AI-animated historical photographs evoke emotional resonance: the mediating role of mental imagery and the moderating effect of perceived historical authenticity

**DOI:** 10.3389/fpsyg.2026.1874146

**Published:** 2026-07-13

**Authors:** Zhang Huanhuan, Li Han, Song Zhangtong

**Affiliations:** School of Creative Design, Wuhan Business University, Wuhan, China

**Keywords:** AI-animated historical photographs, emotional resonance, mental imagery, perceived historical authenticity, presentation format, willingness to share

## Abstract

This study examines how the presentation format of AI-animated historical photographs influences viewers' emotional and communicative responses. Although AI-based animation of historical photographs has become increasingly common in digital historical communication, the psychological mechanism through which dynamic presentation shapes audience engagement remains unclear. Drawing on mental imagery theory, this study proposes that dynamic presentation enhances emotional resonance by facilitating viewers' internal simulation of historical scenes, and that this effect is contingent on perceived historical authenticity. Three experiments were conducted to test this framework. Experiment 1 demonstrated that, compared with static presentation, dynamic presentation significantly increased emotional resonance. Experiment 2 further showed that mental imagery mediated the relationship between presentation format and emotional resonance, whereas emotional state and historical interest did not produce significant mediating effects. Experiment 3 introduced perceived historical authenticity as a moderator and found that the indirect effect of presentation format on emotional resonance through mental imagery was stronger when perceived historical authenticity was high. In addition, emotional resonance significantly predicted willingness to share, and mental imagery and emotional resonance jointly formed a serial mediation pathway linking presentation format to sharing intention. These findings indicate that the effects of AI-animated historical photographs cannot be explained solely by perceptual vividness. Rather, dynamic presentation influences audience response through simulation-based affective processing, and this process depends on whether viewers perceive the content as historically credible. By integrating presentation format, mental imagery, perceived historical authenticity, emotional resonance, and willingness to share into a unified framework, this study advances understanding of how AI-animated historical photographs are emotionally processed and socially circulated.

## 1 Introduction

### 1.1 Research background and problem statement

With the rapid development of AI image restoration and animation technologies, historical photographs have gradually transformed from “static records” into visual content that can be dynamically presented (Tian, 2020). Deep learning algorithms can animate historical photographs by generating subtle movements, such as blinking or smiling, thereby increasing the vividness and immediacy of historical representations. (Palmer 2010) found that animated historical images on social media are more likely to attract viewers' attention, encourage interaction, and stimulate content sharing (Palmer, 2010). Such images may also evoke stronger emotional responses among viewers. However, despite the growing popularity of AI-animated historical photographs, the psychological mechanisms underlying its emotional impact remain insufficiently understood.

Previous research on dynamic visual content has primarily focused on commercial advertising, films, and product promotion videos. These studies consistently demonstrate that motion cues can attract attention and enhance emotional engagement (Green and Brock, 2000). For example Teixeira and others found this out in their research in 2012 (Teixeira et al., 2012). They saw that moving pictures are good at getting people to pay attention and feel emotions. AI-animated historical photographs differ substantially from these contexts. People are using learning algorithms to make old pictures look like they are moving and these moving pictures are, like the ones Palmer talked about. In contrast, moving images in historical contexts carry more complex psychological significance and may involve perceptions of historical authenticity and digital heritage representation (Giaccardi, 2012). Research by Ries et al. indicates that historical photographs are not merely visual stimuli; they also carry individuals' cognitive experiences, emotional memories, and judgments of authenticity regarding historical figures (Ries and Schwan, 2024). Therefore, when these images are animated, the psychological responses they elicit may differ from those triggered by general commercial or entertainment imagery.

Our preliminary data organization and case analysis reveal that dynamic processing often produces two intertwined psychological effects. For example, Okon-Singer et al. (2013) found in empirical research that motion cues enhance a subject's “sense of presence,” making it easier for viewers to form emotional connections. Research by Xu Zhan and others shows that dynamic effects made by algorithms can make people question if the images are real. This can affect how people feel about what they're seeing (Xu and Li, 2014). AI-animated historical photographs are not merely visually enhanced representations of the past. It may simultaneously evoke emotional responses while prompting viewers to evaluate the credibility and authenticity of the content. This dual process is consistent with research on AI-mediated communication, which suggests that audiences often respond not only to media content itself but also to cues regarding technological agency and source credibility (Sundar, 2020). However, the psychological mechanisms underlying these responses remain insufficiently understood.

Among the theoretical perspectives that may explain this phenomenon, Mental Imagery Theory proposed by Finke (1985) provides a particularly relevant framework. This perspective is also consistent with transportation theory, which suggests that richer perceptual cues facilitate psychological immersion and emotional engagement. Mental imagery refers to the process by which individuals internally reconstruct a situation based on perceptual information (Finke, 1985). Existing research indicates that when viewers are able to “fill in” the gaps regarding a character's circumstances and context in their minds, the subjective vividness of the stimulus increases significantly, further amplifying emotional experiences and empathic responses (Wilson et al., 2018; Sutherland, 2015). Compared to static historical photographs, AI-animated historical photographs provide more behavioral cues and temporal information, making it easier to trigger viewers' internal simulation of the subjects' experiences and thereby strengthen emotional resonance (Smith, 1994; Murwonugroho et al., 2025). However, it is worth noting that dynamic visual cues do not consistently produce an emotional enhancement effect in all contexts (Quoidbach et al., 2015). Particularly in historical video contexts, viewers' subjective judgments regarding “whether the footage is authentic” often become a critical factor. Perceived historical authenticity refers to an individual's subjective belief regarding whether the historical photographs faithfully reflect historical figures and their circumstances (Waitt, 2000). Waitt, G. argues that when images are perceived as historically credible, viewers are more likely to become immersed and emotionally engaged; conversely, when dynamic effects are interpreted as “technological manipulation” rather than “historical reenactment,” individuals may maintain psychological distance, and emotional resonance consequently diminishes. Therefore, the perception of authenticity likely plays a moderating role in the process by which dynamic cues influence emotional responses.

While media richness, transportation, and digital heritage perspectives all provide useful insights into audience engagement with historical media, they do not directly explain the cognitive process through which dynamic visual cues are transformed into emotional experiences. To address this gap, the present study adopts Mental Imagery Theory as its primary explanatory framework while incorporating authenticity perception as a key contextual condition.

Based on the above literature and theoretical reasoning, this study attempts to further address the following questions: Why are viewers more likely to experience emotional resonance when old photographs are animated using AI technology? Is this effect achieved through the generation of mental imagery? Under what conditions of perceived historical authenticity is this mechanism most pronounced?

### 1.2 Theoretical framework and hypothesis formulation

#### 1.2.1 Dynamic visual cues and emotional activation: from visual motion to emotional response

In the process of human visual perception, dynamic visual elements tend to attract our attention more easily than static images, a phenomenon long established by research (Raymond, 2000). Dynamic content is so eye-catching not only because it is visually more striking, but more importantly, because this temporal dimension of change imbues the image with a vivid sense of vitality (Pei et al., 2022). From a psychological perspective, people habitually associate movement with life. When we see things moving it usually means there is life and activity (Röcke et al., 2023). Movement also gives us a chance to interact with others. For example when we look at moving pictures or objects our brain automatically gives them meaning and thinks about what they might do like they are alive. This happens because our brain is used to seeing movement as a sign of something like danger or a good thing, which helped our ancestors survive. The human visual system is very good, at detecting movement. This is because in nature movement often signals something we need to pay attention to, like movement of the movement.

From the perspective of associative learning, individuals gradually form stable perceptual-emotional associations through long-term experience (Gendron and Barrett, 2018). Research by Van Kleef et al. (2015) indicates that when certain cues reappear, the associated emotional representations are automatically activated and influence attitudes and evaluations. Existing research in advertising and media communication contexts has repeatedly found that dynamic visual cues can significantly enhance emotional arousal and psychological engagement (Yang et al., 2025). However, this mechanism may not operate identically in historical imagery contexts. This difference may be understood through the perspectives of Media Richness Theory and digital heritage studies. Media Richness Theory suggests that communication formats containing richer sensory information are more likely to facilitate engagement and emotional involvement (Daft and Lengel, 1986). In the context of historical imagery, dynamic presentation provides additional temporal and behavioral cues that may increase the perceived immediacy of historical figures and events. Furthermore, digital heritage research emphasizes that technologically enhanced representations of the past can strengthen audiences' emotional connections with historical subjects by making historical experiences appear more vivid and accessible (Ries and Schwan, 2024). Historical photographs inherently carry temporal distance and memory attributes; their meaning derives not only from visual information but also from individuals' cognitive interpretations of the “past” (Crane, 2013). From the perspective of digital heritage studies, technologically enhanced representations of historical content may help audiences experience the past in a more vivid and accessible manner, thereby strengthening emotional engagement with historical subjects (Champion, 2016). Consequently, when static historical figures are imbued with subtle movements, viewers are no longer confronted merely with a “record” but rather with a social context that appears to be “unfolding.” When static historical figures are imbued with subtle movements, viewers are no longer confronted merely with a “record” but rather with a social context that appears to be “unfolding.” This shift may reactivate individuals' existing experiences of life and interaction, leading them to perceive historical figures as individuals with emotions and life experiences, thereby facilitating emotional connection and empathy.

Based on the above reasoning, this study posits that in the context of AI-animated historical photographs, dynamic presentation not only enhances visual vividness but may also strengthen viewers' emotional resonance by activating emotional connection structures related to life experiences. Therefore, we propose:

H1: Compared to static historical photographs, dynamically presented AI-animated historical photographs can significantly increase viewers' emotional resonance.

#### 1.2.2 The mediating role of mental imagery: how visual stimuli are transformed into emotional experiences

(Atkinson and Adolphs 2005) argue that while dynamic images often elicit emotional responses more readily, visual stimuli themselves do not directly equate to emotional experiences; their effects typically require internal psychological processing (Atkinson and Adolphs, 2005). Although alternative perspectives such as Media Richness Theory and Narrative Transportation Theory can help explain why audiences become engaged with mediated experiences, these frameworks primarily focus on the richness of media information or the immersive state experienced during media consumption (Green and Brock, 2000). In contrast, Mental Imagery Theory provides a more direct explanation of the psychological mechanism through which visual cues are translated into emotional responses. Specifically, mental imagery emphasizes the internal reconstruction of situations, characters, and experiences based on perceptual information. Because the present study aims to explain how AI-animated historical photographs evoke emotional resonance, the theory is particularly suitable for identifying the cognitive process that links dynamic visual cues to emotional outcomes. Therefore, Media Richness Theory and Transportation Theory are treated as complementary perspectives that explain audience engagement, whereas Mental Imagery Theory serves as the primary explanatory framework for the mediating mechanism examined in this study.

Mental imagery is not merely a simple visual reproduction, but rather a process of internally constructing characters and situations based on perceptual information (Pylyshyn, 2002). That is, when individuals form situational experiences in their minds, their emotional responses tend to be more intense and persistent. Research by Shin (2018) and Smith et al. (2012) indicate that visual content capable of stimulating mental imagery processing is more likely to evoke immersive experiences and empathetic responses. Compared to static images, dynamic images provide temporal cues and behavioral changes, making it easier for viewers to internally establish situational continuity, thereby enhancing the completeness of mental simulation (Shin, 2018; Smith et al., 2012). In the context of AI-animated historical photographs, this mechanism may be even more pronounced. Research by Singh and Malik (2025) indicates that subtle movements and facial expressions of subjects provide viewers with behavioral cues for inference, enabling them to automatically fill in the emotional states and life contexts of the subjects during viewing (Singh and Malik, 2025). Furthermore, research by Davis et al. (2011) also indicates that dynamic presentation shortens the psychological distance between historical imagery and real-life experience, making it easier to perceive “people from the past” as “people currently experiencing a particular situation” (Davis et al., 2011). When this internal simulation is activated, viewers not only comprehend the content of the footage but may also experience a sense of immersion on an emotional level, thereby enhancing emotional resonance.

Based on this, this study posits that dynamic presentation may first influence emotional experience by enhancing the level of mental imagery. Consequently, we propose:

H2: Mental imagery mediates the relationship between the presentation format of AI-animated historical photographs and emotional resonance.

#### 1.2.3 The moderating role of perceived historical authenticity: authenticity as a key boundary condition

It is important to note that, as pointed out in the study by Chen and Liu (2016), dynamic visual cues do not consistently produce emotion-enhancing effects in all contexts. Particularly in the context of historical imagery, viewers' subjective judgments regarding the authenticity of the images may become a critical factor. Perceived historical authenticity does not refer to whether the imagery is objectively accurate, but rather to the subjective experience of “credibility” formed by the individual during the viewing process (Chen and Liu, 2016). This conceptualization is consistent with prior authenticity research, which argues that audiences often respond to subjective perceptions of credibility rather than objective historical accuracy (Waitt, 2000; Stepchenkova and Park, 2021). Consequently, perceived authenticity may function as an important psychological boundary condition influencing emotional engagement with AI-animated historical media.

Research by Sundar et al. (2017) indicates that when media content is perceived as authentic, viewers are more likely to experience immersion and emotional resonance; conversely, when content is perceived as overly processed or artificially constructed, individuals tend to maintain psychological distance, thereby reducing emotional resonance (Baños et al., 2004). In AI-animated historical photographs, this judgment is particularly pronounced. Motion processing can either enhance the sense of life in characters or trigger cognitive awareness of “technological generation” (Zhang N. et al., 2024). Therefore, in the context of historical footage, the perception of authenticity is likely to serve as a critical boundary condition for dynamic emotional effects. When viewers perceive an image as having a high degree of perceived historical authenticity, dynamic cues are more easily integrated into their historical cognitive framework, thereby promoting the generation of mental imagery and enhancing emotional resonance; conversely, when authenticity is low, viewers may maintain a cognitive distance from the image, limiting emotional resonance.

Based on this, we propose:

H3: Perceived historical authenticity moderates the effect of presentation format on mental imagery; under high-authenticity conditions, dynamic presentation has a stronger promotional effect on mental imagery.

H4: Perceived historical authenticity moderates the effect of presentation format on emotional resonance; under conditions of high authenticity, dynamic presentation has a more significant enhancing effect on emotional resonance.

H5: Perceived historical authenticity significantly moderates the mediating role of mental imagery in the process by which presentation format influences emotional resonance.

#### 1.2.4 Emotional resonance and communication behavior: a chain-like psychological pathway

Emotions are not only the result of an individual's internal experience but also often serve as a key driver of behavior. Research by Giorgi (2017) indicates that when individuals experience strong emotional resonance, they are more likely to prolong that emotional experience through sharing and recommendations. In digital media environments, emotional drive has been recognized as one of the key mechanisms underlying the spontaneous dissemination of content (Hasell and Nabi, 2023). In the context of dynamically presented AI-animated historical photographs, the dynamic presentation may first activate mental imagery through visual motion cues, enabling viewers to internally reconstruct characters and scenarios; subsequently, this mental imagery reinforces emotional experiences and fosters emotional resonance; once emotional resonance reaches a certain intensity, individuals are more likely to sustain this emotional state through sharing behaviors.

Based on this logic, this study further proposes:

H6: Mental imagery and emotional resonance play a chained mediating role in the process by which presentation format influences the willingness to share.

### 1.3 Research model

**Figure 1:**
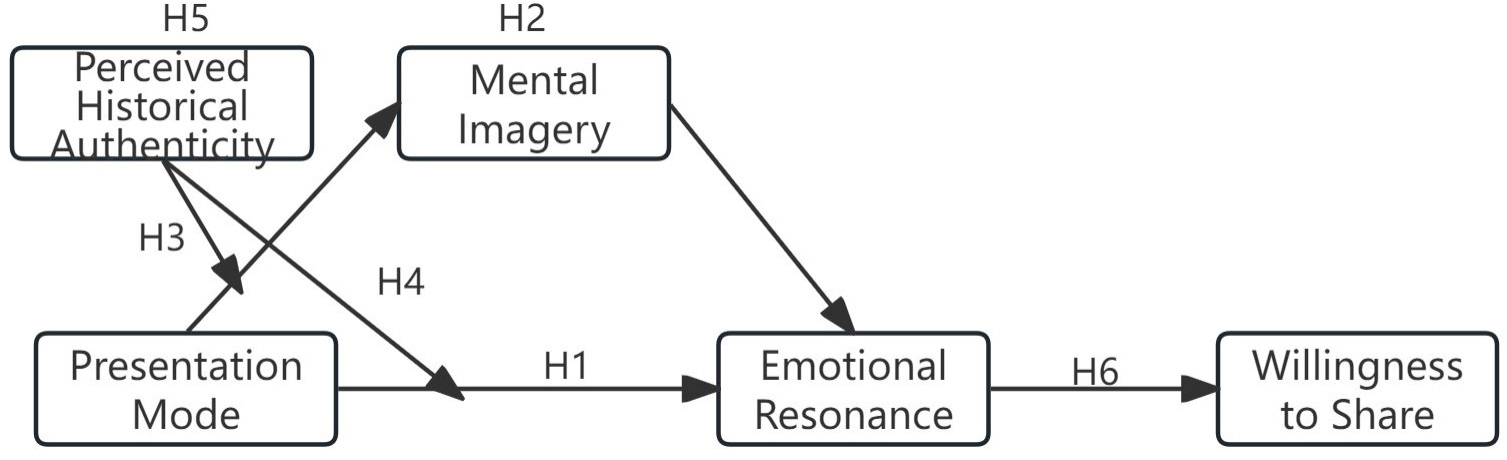
Proposed research model.

To systematically examine the psychological mechanisms underlying how AI-animated historical photographs influence audience emotional responses and sharing behavior, this study integrates insights from associative learning theory, mental imagery theory, authenticity perception research, and related perspectives on media richness and digital heritage communication. We attempt to construct a comprehensive model of “presentation mode—mental imagery—emotional resonance—willingness to share” (see Figure 1) by integrating the perspectives of associative learning theory, mental imagery theory, and the perception of authenticity. In this model, AI-animated historical photographs are viewed as a visual stimulus with the potential to evoke emotions; however, its influence does not act directly on emotional responses but is realized gradually through internal psychological processing. We predict that dynamic presentation first prompts viewers to internally construct characters and contexts through motion cues, thereby forming mental imagery; this mental imagery then further intensifies emotional experiences, manifesting as more pronounced emotional resonance; once emotional resonance is established, individuals are more likely to prolong their emotional experiences through sharing behaviors. Concurrently, our model identifies the perception of perceived historical authenticity as a key contextual variable. Unlike the visual stimulus itself, this variable reflects the audience's subjective understanding. When images are perceived as having high perceived historical authenticity, viewers are more likely to integrate them into existing cognitive frameworks, thereby enhancing the generation of mental imagery; conversely, when perceived historical authenticity is low, individuals may maintain a certain cognitive distance, limiting mental simulation and emotional resonance. Thus, perceived historical authenticity constitutes a critical boundary condition for the emotional effects of AI-animated historical photographs. Based on the above logic, this study constructs a path model integrating visual presentation features, psychological processing, and communication behavior. Through three sequential experiments, the study seeks to explain how AI-animated historical photographs transform from perceptual stimuli into emotional experiences and further influences communication behavior, thereby providing empirical support and theoretical contributions to research on visual emotional processing and generative AI media psychology.

## 2 Methods

### 2.1 Overall research design

To systematically examine the psychological mechanisms underlying how the presentation format of AI-animated historical photographs affects audience emotional responses and communication behavior, we conducted three sequential experiments, progressing logically from “main effect testing—mediation mechanisms—situational moderation and behavioral outcomes.” Experiment 1 employed a single-factor two-level between-subjects design to compare dynamic vs. static presentation formats on emotional resonance. Experiment 2 extended this design by introducing mental imagery as a mediating variable, thereby examining the psychological pathway through which presentation format influences affective outcomes. Experiment 3 adopted a 2 × 2 between-subjects design, systematically manipulating both presentation format and perceived historical authenticity, while additionally assessing willingness to share as a behavioral outcome. We also looked at how willing people're to share. All experiments were conducted online using a standardized survey platform, and participants were randomly assigned to experimental conditions. Although online experiments enhance ecological validity and accessibility, they may also introduce uncontrolled environmental influences. Therefore, standardized instructions and quality-control procedures were implemented throughout the study.

We used SPSS 26.0 and the PROCESS macro, for analysis. All experimental procedures adhered to psychological experimental standards and were executed under the same technical environment and presentation parameters to ensure comparability and internal validity across experiments.

Participants were recruited through the university's online research participation system and campus-wide recruitment announcements. Eligibility criteria required participants to be undergraduate or graduate students aged 18 years or older with normal or corrected-to-normal vision. Participation was voluntary, and students received either course credit.

### 2.2 Stimuli

The experimental stimuli consisted of historical photographs selected from public-domain historical image archives and used solely for academic research. Twelve photographs were initially screened, and a pilot study (*n* = 50) was conducted to assess familiarity, emotional valence, and arousal, ensuring no significant differences in basic emotional attributes among the materials. Ultimately, four photographs showing no significant differences across the aforementioned dimensions were selected as the formal experimental materials. To control for visual interference variables, all images underwent uniform resolution standardization, size normalization, and fine-tuning of lighting and contrast (while preserving their historical texture) prior to the formal experiment. This was done to ensure that experimental manipulation altered only the “presentation format” without introducing additional visual biases.

### 2.3 AI animation procedure for historical photographs

To ensure the technical transparency and reproducibility of the stimulus materials, this study standardized the AI dynamic generation process. (1) The generation tool used was the Dreamina AI image animation generation system. The input material consisted of digitized high-resolution historical portraits, and the animation type was facial micro-motion generation (blinking, smiling, and slight head movements). To maintain consistency across stimuli, all animations were restricted to subtle facial movements, including eye blinking, slight smiling, and minimal head movement. No large-scale body movement, scene transition, or additional visual effects were introduced. (2) Animation principles require that the facial structure of the subjects remain unchanged, the original historical texture and lighting be preserved, no background or modern elements be added, and no style transfer be performed. The output format is MP4, with a duration of 8 s. (3) Researchers conduct a double manual review to exclude samples with abnormal movements or facial distortion, ensuring natural motion amplitude. Under the static condition, the original historical photographs were presented without animation, with dimensions, resolution, and display duration consistent with those under dynamic conditions. This process aims to ensure that the experimental manipulation is reflected solely in the “presence or absence of dynamic cues,” rather than other visual differences. In the present study, the term AI-animated historical photographs refers to historical photographs that were digitally animated using AI-based image animation techniques while preserving the original visual content and historical appearance. The study focuses on the effects of animation rather than image restoration or AI image generation.

To make sure we can repeat the results we used pictures from historical archives like the Library of Congress digital collections and other open historical image repositories. We only used pictures that're free for anyone to use. For the animation part we used Dreamina AI, with the settings every time. We gave the instructions for every picture: “Make the face move a little like blinking or a small smile and move the head a bit but keep the face and the background looking the same, as the original with the same lighting and texture and make sure it does not look too modern or fake.” We had two people check all the animated pictures to make sure they looked okay. We did not use the ones that looked distorted had too much movement or did not look like the original face.

### 2.4 Experimental procedure

The experiment was conducted via an online experimental system. First, participants logged into the system and read the informed consent statement; second, the system randomly assigned them to an experimental condition; third, participants viewed four images in sequence (each displayed for 8 s), with the order randomized; fourth, after viewing the images, participants completed scales measuring emotional resonance, mental imagery (Experiments 2 and 3), emotional state, historical interest, and willingness to share (Experiment 3); finally, demographic information was collected. The entire experiment takes approximately 8–10 min.

Random assignment was implemented automatically by the online experimental platform. After providing informed consent, participants were assigned to experimental conditions using a computer-generated randomization algorithm with an equal allocation ratio across groups.

All experiments were administered through WenJuanXing (URL: https://www.wjx.cn/), a widely used online survey and experimental platform in China. The platform supported random assignment, stimulus presentation, response recording, and data export.

### 2.5 Exclusion criteria

To ensure data quality, cases where participants did not complete the entire experimental procedure, had reaction times below one-third of the sample median or above three times the standard deviation, provided consecutive responses to the same option (linear response pattern), or had more than 20% missing data were excluded before the sample was subjected to statistical analysis.

### 2.6 Measurement instruments

All scales use a 7-point rating scale (1 = Strongly Disagree, 7 = Strongly Agree). The scales include: emotional Resonance, Mental Imagery, Perceived historical authenticity, Emotional State, and Willingness to Share. Each scale is derived from existing psychological research and has been appropriately revised for the context of this study to ensure measurement validity. Furthermore, the scales were adapted from established scales in prior research. To ensure suitability for the historical imagery context, item wording was revised while preserving the original construct definitions. The adapted scales were reviewed by three experts in communication and psychology and were pilot tested prior to formal data collection. Reliability analyses demonstrated satisfactory internal consistency across all measures (α > 0.90).

### 2.7 Data analysis strategy

All data were analyzed using SPSS 26.0. Prior to hypothesis testing, assumptions of normality, homogeneity of variance, and linearity were examined. No substantial violations were detected. Main effects were analyzed using ANOVA/ANCOVA; mediation effects were analyzed using PROCESS Model 4; moderation effects were analyzed using PROCESS Model 8; and chained mediation was analyzed using PROCESS Model 6. Bootstrap sampling was conducted 5,000 times, and 95% confidence intervals were reported. The significance level was set at α = 0.05.

We conducted Harman's single-factor test to assess the potential influence of common method variance. The results indicated that the first unrotated factor accounted for less than 40% of the total variance, suggesting that common method bias was unlikely to pose a serious threat to the validity of the findings.

### 2.8 Ethics statement

This study was approved by the Academic Ethics Committee, School of Creative Design, Wuhan Business University. All participants provided written informed consent prior to participation. Personal data were treated as strictly confidential and used solely for research purposes. All procedures were conducted in accordance with the ethical principles outlined in the Declaration of Helsinki, ensuring the protection of participants' rights, dignity, and welfare.

### 2.9 Data availability

Research data are available upon reasonable request from the corresponding author.

## 3 Experiment 1: the effect of AI-animated historical photograph presentation format (dynamic vs. static) on emotional resonance

Experiment 1 aims to examine whether the presentation format (dynamic/static) of AI-animated historical photographs has a significant impact on viewers' levels of emotional resonance, thereby providing foundational evidence for subsequent investigations into the mechanisms of psychological and regulatory effects.

### 3.1 Experimental design and procedure

#### 3.1.1 Participants

A total of 197 college students were recruited for this experiment (96 males, 101 females), aged 18–24 (M = 20.69, SD = 1.89). All participants were right-handed and had normal vision or corrected vision. A prior power analysis was conducted using G^*^Power. Under a two-group one-way design with an effect size of *f* = 0.25 and a significance level of α = 0.05, a sample size of 197 corresponded to a statistical power of 0.936, meeting the study requirements. Participants voluntarily enrolled through the campus experiment platform and received course credit upon completion of the experiment. All participants signed informed consent forms prior to the experiment. This study employed a two-level between-subjects design with a single independent variable: presentation format (dynamic vs. static), and the dependent variable was emotional resonance. After random assignment, 99 participants were assigned to the dynamic group and 98 to the static group.

#### 3.1.2 Experimental procedures

Four historical photographs were selected as the original experimental stimuli from public-domain digital archives. Prior to the main experiment, a pilot study (*n* = 50) was conducted to assess participants' familiarity with and affective responses to each image, confirming the absence of significant between-image differences in baseline emotional valence and arousal. The results showed that the pictures were much the same when it came to how people felt about them.

In the dynamic condition, the historical photographs were transformed into AI-animated historical photographs using Dreamina AI, so it looked like the people in the pictures were blinking, smiling and moving their heads a bit. The original picture was not changed it still looked old. The faces were still the same. Each AI-animated historical photographs was shown for 8 s. In the static condition, participants viewed the original historical photographs without animation and they looked at them for the same amount of time.

People started the test by logging into a website and agreeing to be part of it. Then they were put into one of the two groups either the moving picture group or the still picture group. After they looked at the pictures they answered some questions about how they felt, using something called the Emotional Resonance Scale and the Emotional State Scale. At the end they were asked some questions, about themselves like how old they are. The whole thing took 10 min to finish.

#### 3.1.3 Measurement instruments

In this experiment, participants were asked to complete the Emotional Resonance Scale. The Emotional Resonance Scale used in this study is an adapted version of the scale developed by Batson et al. (1988) and Zillmann and Cantor (1977). Because the original scales were developed in non-historical contexts, item wording was modified to fit the viewing experience of historical photographs. The adaptation focused on replacing general media references with references to historical figures and historical scenes while preserving the original conceptual meaning.

The scale is consisting of four items: “Looking at this photo touches me deeply,” “I seem to be able to sense the emotions of the person in the photo,” and “I feel an emotional connection with the person in the photo” (α = 0.97). On this scale 1 means you strongly disagree and 7 means you strongly agree. Higher scores show an emotional connection. The experiment made sure to consider peoples feelings and interests. It checked how people were feeling using a short mood test created by Watson, D. And others in 1988 (Watson et al., 1988). This test had three questions: “I currently feel happy,” “I currently feel relaxed and comfortable.”

CI currently feel relatively positive” (α = 0.914). Each of these questions used a scale where 1 means strongly disagree and 7 means strongly agree. It also measured how much people are interested in history with one question: “I have a high level of personal interest in history-related content.” This question was rated on a 7-point scale, where 1 means not all interested and 7 means very interested. Then people provided some information, about themselves like their age and gender.

### 3.2 Results

#### 3.2.1 Randomization and sample balance tests

Statistical analysis was conducted on the valid sample. No significant differences were found between the dynamic group and the static group in terms of gender ratio (χ^2^ = 0.005, *p* = 0.94), age (*t*_(195)_ = 0.368, *p* = 0.71), level of historical interest (*t*_(195)_ = 0.766, *p* = 0.45), emotional state (*t*_(195)_ = 1.41, *p* = 0.16). This indicates that random assignment was successful and that the groups are comparable.

#### 3.2.2 Assumption testing

We were prior to conducting the ANOVA, the assumptions of normality and homogeneity of variance were examined. Visual inspection of the distributions and residual plots indicated no substantial deviations from normality. Levene's test showed that the homogeneity of variance assumption was satisfied (*p* > 0.05). Therefore, the data met the assumptions required for ANOVA.

#### 3.2.3 Analysis of main effects

To examine the effect of presentation format on emotional resonance, a one-way analysis of variance (ANOVA) was conducted, with the presentation format of AI-animated historical photographs (dynamic/static) as the independent variable and emotional resonance as the dependent variable. The results showed that presentation format had a significant main effect on emotional resonance, *F*_(1, 195)_ = 16.447, *p* < 0.001, ηp^2^ = 0.078. According to Cohen's (1988) guidelines, this represents a medium effect size. Specifically, emotional resonance in the dynamic group (M = 5.68, SD = 0.85) was significantly higher than in the static group (M = 5.17, SD = 0.93). This effect remained significant after controlling for emotional state and historical interest (*F*_(1, 193)_ = 16.482, *p* < 0.001, ηp^2^ = 0.08). The magnitude of this effect remained within the medium effect size range. The results remained stable after further controlling for demographic variables (gender, age; *F*_(1, 191)_ = 16.30, *p* < 0.001, ηp^2^ = 0.08), indicating robustness across covariate adjustments.

### 3.3 Interpretation of results and theoretical implications

The results of Experiment 1 show that AI-animated historical photographs evoke significantly higher levels of emotional resonance than static historical photographs have a much bigger impact on peoples emotions than static presentations. This is true even when we take into account how people are feeling how interested they are in history and other personal factors. So it seems that the way the photographs are presented is what really matters, not the differences between people. From a point of view this result supports the idea that we can use associative learning theory to understand how people react to historical images. When we see visual cues it reminds us of things that are connected to being alive interacting with others and being in certain situations. This makes it easier for us to see people in photographs as real people with feelings and experiences which helps us feel more connected to them and understand them better as Murwonugroho et al. (2025). Also these results tell us that visual stimuli, in historical images do not just affect us because they are more vivid but because they make our minds work in a deeper way. The dynamic cues might make us imagine situations clearly in our minds, which makes us feel more emotion.

Therefore, Experiment 1 not only validated Hypothesis 1 but also provided a key theoretical clue for subsequent research: dynamic presentation may influence emotional experiences by activating internal mental representations. Based on this inference, Experiment 2 will further introduce the mental imagery variable to test its mediating role between presentation format and emotional resonance, thereby constructing a more comprehensive model of the underlying psychological mechanisms.

## 4 Experiment 2: the mediating role of mental imagery

The results of Experiment 1 indicate that dynamically presented AI-animated historical photographs significantly enhance viewers' emotional resonance. However, the underlying psychological mechanisms of this effect have not yet been directly examined. According to mental imagery theory, visual stimuli typically influence emotional experiences through internal scenario simulation; thus, dynamic presentation may enhance emotional resonance by activating mental imagery. To test this hypothesis, Experiment 2 introduced mental imagery as a mediating variable, building upon the findings of Experiment 1, to systematically explore the psychological processing pathways through which presentation format influences emotional resonance.

### 4.1 Experimental design and procedure

#### 4.1.1 Participants

The study had 212 college students. These students were 103 males and 109 females. They were all between 18 and 25 years old. On average they were 21.46 years old. All the students who took part in the study were right-handed. They all had eyesight or wore glasses to correct their eyesight. None of the students, in Experiment 2 had any problems or issues with their vision. These students volunteered to be in the study. They did this through the campus experiment recruitment platform. When they finished the study they got course credits or a little bit of money. A prior power analysis was conducted using G^*^Power. Under a two-group design, with an effect size of *f* = 0.25, a significance level of α = 0.05, and a statistical power of 1–β = 0.90, the minimum sample size was calculated to be 180. The actual sample size for this study was 212, with a statistical power of 0.95, meeting the study requirements. This experiment employed a one-factor, two-level between-groups design, with the independent variable being the presentation format of AI-animated historical photographs (dynamic/static), the dependent variable being emotional resonance, and the mediating variable being mental imagery. The students were split into two groups. One group got the moving photographs. The other group got the still photographs. There were 106 students, in each group. Before the study started all the students agreed to take part. When they finished the study they got course credits or a little bit of money.

#### 4.1.2 Materials and procedure

The pictures we used for this test were the same as the ones we used in the test. We picked four pictures of famous people from public picture websites and only used them to learn more about this topic. We wanted to make sure the two tests were similar so we made sure all the pictures had the feeling were just as familiar and got the same reaction from people. We did a test before the main test and found that the pictures were all pretty much the same. When we showed the pictures in a lively way we used a special computer program called DreaminaAI to make the pictures move a little bit. This program added movements to the people, in the pictures like blinking, smiling and moving their heads slightly. Each clip lasted approximately 8 s, with movement amplitude kept within natural limits to avoid interference from exaggerated effects. Under the static presentation condition, participants viewed the original historical photographs of the same subjects. To minimize the interference of authenticity expectations on the results, the experimental instructions did not emphasize that the images had been animated using AI technology; participants were only informed that the study aimed to explore the viewing experience of different historical image presentation formats; participants were only informed that the study aimed to explore the viewing experience of different historical image presentation formats. The experiment was conducted via an online experimental system, with an overall process largely consistent with Experiment 1, lasting approximately 10 minutes: (1) Participants logged into the experimental system and read the informed consent statement; (2) The system randomly assigned participants to either the dynamic or static presentation condition; (3) Participants viewed four images of historical figures sequentially (each displayed for 8 s); (4) After all images were presented, participants completed the Mental Imagery Scale and the Emotional Resonance Scale sequentially; (5) Participants completed questionnaires regarding their emotional state, historical interest, and demographic information; (6) The order of all image presentations was randomized to minimize the interference of order effects.

#### 4.1.3 Measurement instruments

In this experiment, participants were asked to complete the Mental Imagery Scale, adapted from the Visual Mental Imagery Scale by MacInnis and Price (1987) and D'Ercole et al. (2010). The original mental imagery items were adapted to reflect imagery elicited by historical photographs. All items were reviewed by two communication scholars and one psychology researcher to ensure content validity.

The scale consists of 5 items, such as “I can clearly ‘see' the figure in the photograph in my mind,” “I can imagine how the people in the photograph would act or speak in that situation,” “When looking at this photograph, related images come to mind,” “I feel as if I have ‘entered' the scene in the photograph,” and “I can sense the atmosphere of the scene in the photograph” (α = 0.93). Responses were rated on a 7-point scale, where 1 represents “Strongly Disagree” and 7 represents “Strongly Agree.” In addition, participants were required to complete the Emotional Resonance Scale, the Emotional State Scale, and the Historical Interest Scale (a self-developed single-item scale), with items identical to those in Experiment 1. The Emotional Resonance Scale had a reliability coefficient (α) of 0.91, and the Emotional State Scale had an α of 0.91.

### 4.2 Results analysis

#### 4.2.1 Assumption testing

Prior to conducting mediation analyses, assumptions of normality, homoscedasticity, and linearity were examined. Residual distributions approximated normality, and no evidence of severe multicollinearity was observed among the study variables (all VIFs < 5). Therefore, the assumptions for regression-based mediation analysis were satisfied.

#### 4.2.2 Analysis of participant demographics

After checking the questionnaires for missing information and weird response times we were left with 208 samples. (106 were in the dynamic group and 102 were in the static group). The response rate was 98.1%. The two groups differed in terms of the presentation format of AI-animated historical photographs regarding gender (χ^2^ = 0.08, *p* = 0.78), age (*t*_(206)_ = 0.57, *p* = 0.57), emotional state (*t*_(206)_ = 1.20, *p* = 0.23), and historical interest (*t*_(206)_ = 0.25, *p* = 0.81). This shows that the groups were randomly assigned correctly.

#### 4.2.3 Re-examination of main effects

To replicate the findings of Experiment 1, an ANCOVA was conducted to examine whether presentation format (dynamic vs. static) significantly influenced emotional resonance. The people doing the experiment wanted to know if the presentation format affects the resonance. They made sure to consider the state and historical interest of the people in the experiment. The results showed that presentation format had a significant effect on emotional resonance, *F*_(1, 204)_ = 26.34, *p* < 0.001, ηp^2^ = 0.11;Specifically, the dynamic group (M = 5.31, SD = 0.66) scored higher than the static group (M = 4.80, SD = 0.77). After controlling for demographic variables such as gender and age, the results remained significant (*F*_(1, 202)_ = 24.83, *p* < 0.001, ηp^2^ = 0.11). This effect can be interpreted as a medium-to-large effect according to Cohen's (1988) benchmarks. These results are similar, to what was found in Experiment 1 which means that we can trust that dynamic presentations really do affect resonance.

#### 4.2.4 Testing for mediating effects

First, we examined how the presentation format of AI-animated historical photographs, which is either static or dynamic affects imagery. We also considered how people feel and how interested they are in history. The results showed that the presentation format (static/dynamic) had a significant effect on mental imagery, *F*_(1, 204)_ = 12.91, *p* < 0.001, ηp^2^ = 0.06. Mental imagery in the dynamic group (M = 5.79, SD = 0.73) were significantly higher than those in the static group (M = 5.38, SD = 0.91). After controlling for demographic variables such as gender and age, the effect of presentation format (static/dynamic) on mental imagery remained significant, *F*_(1, 202)_ = 12.14, *p* = 0.001, ηp^2^ = 0.06.

The researchers then examined how mental imagery influences emotional resonance when viewers observe AI-animated historical photographs. They used a model to test this called PROCESS Model 4 and they did this test 5,000 times. The researchers wanted to see how the way the photographs were presented either in an dynamic way affected peoples emotional resonance. They also looked at how mental imagery and peoples historical interest affected this relationship. The results showed that mental imagery significantly mediated the effect of presentation format (static/dynamic) on emotional resonance (β = 0.19; 95% CI [0.08, 0.31]). The indirect effect was considered statistically significant, because the confidence interval did not include zero. Specifically, the effect of presentation format on mental imagery was significant (β = 0.41, SE = 0.11, *p* < 0.001);the effect of mental imagery on emotional resonance was significant (β = 0.47, SE = 0.05, *p* < 0.001); and the direct effect of presentation format on emotional resonance remained significant (β = 0.32, SE = 0.09, *p* = 0.003), indicating partial mediation. After controlling for demographic variables such as gender and age, the mediating effect of mental imagery in the relationship between presentation format and emotional resonance remained significant (β = 0.18; 95% CI [0.08, 0.31]). The confidence interval excluded zero, further supporting the robustness of the mediation effect. The researchers think that mental imagery helps to explain why people feel resonance when they see these photographs and this is true even when they look at the photographs, in different ways.

#### 4.2.5 Test of competitive mediation

To see if the way something is presented affects how people feel and what they are interested in a special test called a one-way ANOVA was done. The test looked at how presentation format, which is either static or dynamic affects emotional state and historical interest. The results showed that presentation format (static/dynamic) had no significant effect on emotional state (*F*_(1, 206)_ = 1.43, *p* = 0.23), nor did presentation format (static/dynamic) have a significant effect on historical interest (*F*_(1, 206)_ = 0.06, *p* = 0.81). Specifically, there was no significant difference in emotional state between the dynamic presentation group (M = 4.37, SD = 1.10) and the emotional state in the static presentation group (M = 4.55, SD = 1.00) showed no significant difference. Furthermore, there was no significant difference in historical interest between the dynamic presentation group (M = 4.15, SD = 1.00) and the static presentation group (M = 4.19, SD = 1.08). Furthermore, we conducted a parallel mediation analysis using emotional state, historical interest, and mental imagery as mediating variables. The results showed that the mediating effect of emotional state was not significant (β = −0.001; 95% CI [−0.02, 0.02]), nor was the mediating effect of historical interest (β = 0.001; 95% CI [−0.01, 0.02]), but the mediating effect of mental imagery remained significant (β = 0.19; 95% CI [0.08, 0.32]). Therefore, this experiment can rule out the alternative mediating roles of emotional state and historical interest, indicating that the emotional advantage of dynamic presentation primarily stems from the activation of mental imagery, rather than differences in immediate emotions or historical interests.

These results provide evidence for the discriminant validity of the mediation model: emotional state and historical interest, despite being plausible alternative mediators, did not account for significant variance in emotional resonance beyond that explained by presentation format. Multicollinearity diagnostics indicated acceptable VIF values for all predictors (VIFs < 2.0), suggesting no problematic collinearity among the three proposed mediators.

### 4.3 Theoretical interpretation and implications

The results of Experiment 2 further reveal that dynamic presentation does not directly enhance emotional resonance but rather exerts its effect through the internal processing of mental imagery. This finding supports the central role of mental imagery theory in visual emotion processing. Dynamic visual cues provide viewers with temporal continuity and behavioral information, making it easier for them to construct internal mental simulations of the characters' situations and emotional states (De Koning and Tabbers, 2011). When individuals “enter” the visual scenario on a psychological level, their emotional responses are more intense and sustained, leading to higher levels of emotional resonance. Furthermore, this mechanism is not driven by immediate emotions or historical interest but primarily stems from the process of mental imagery formation. This suggests that the emotional advantage of AI-animated historical photographs arises from internal mental simulation rather than mere visual vividness or individual differences. Therefore, Experiment 2 not only validated Hypothesis 2 but also clarified, at the mechanistic level, the psychological pathway through which dynamic visual stimuli influence emotional experience: visual motion cues → mental imagery → emotional resonance. This result provides a theoretical foundation for the subsequent introduction of the authenticity variable. Since the generation of mental imagery depends on the audience's cognitive acceptance of the images, the perceived historical authenticity may play a key moderating role in this process. Based on this, we further tested the moderating role of perceived historical authenticity in this psychological pathway through Experiment 3 and constructed a more comprehensive model of the mechanism.

## 5 Experiment 3: the moderating role of perceived historical authenticity and the chain mediation mechanism

Through the first two experiments, we have verified at both the overall effect and psychological mechanism levels that the dynamic presentation of AI-animated historical photographs can significantly enhance viewers' emotional resonance, with this effect mediated by mental imagery. However, the emotional effects of dynamic visual cues may not be consistently present in all contexts. Particularly in the context of historical imagery, viewers' judgments regarding the authenticity of the images may significantly influence their cognitive processing. Therefore, Experiment 3 further introduced perceived historical authenticity as a contextual moderator to examine its role in the “presentation format—mental imagery—emotional resonance” pathway, and on this basis, to explore the chained effects of mental imagery and emotional resonance on the willingness to share.

### 5.1 Pilot study: validation of the manipulation of perceived historical authenticity

Prior to the main experiment, a pilot study was conducted to screen stimulus materials and ensure the validity of the perceived historical authenticity manipulation. A total of 80 college students were recruited for the pilot study, including 38 males and 42 females, with a mean age of 21.33 years (SD = 2.4). None of the participants had taken part in Experiments 1 or 2. Participants were randomly assigned to either the high-authenticity group or the low-authenticity group.

The experimental materials consisted of eight old photographs of historical figures, for which two versions of the historical photographs were created: the high perceived historical authenticity version required the preservation of the original graininess, light and shadow structures, historical noise, and texture; the low perceived historical authenticity version required the use of deep restoration techniques to excessively smooth the images and enhance modern colors, thereby significantly reducing the historical texture. For the high-authenticity condition, historical texture, film grain, lighting artifacts, and age-related visual characteristics were retained. For the low-authenticity condition, images were extensively enhanced through smoothing, colorization, and texture removal, resulting in a more modernized appearance.

Participants viewed 4 randomly presented images and completed the perceived historical authenticity Scale (Stepchenkova and Park, 2021), which consists of 5 items, including “These images convey a sense of perceived historical authenticity,” “I believe the scenes in the photos actually happened,” “I can sense the authentic historical context behind the images,” “These photos look like genuine historical records,” and “The images as a whole convey a reliable sense of perceived historical authenticity” (α = 0.97), with 1 representing “Strongly Disagree” and 7 representing “Strongly Agree.”

Analysis using an independent samples *t*-test revealed that the scores for photos with high perceived historical authenticity (M = 5.93, SD = 0.63) were significantly higher than those for photos with low perceived historical authenticity (M = 4.04, SD = 0.92; *t*_(78)_ = 10.75, *p* < 0.001, Cohen's *d* = 2.40), indicating that the manipulation method used in this experiment was effective and can be adopted as a standard procedure for formal experiments.

### 5.2 Main experiment

#### 5.2.1 Participants

A total of 221 college students took part in the experiment. Out of these 117 were males making up 52.9% of the group. The average age of the students was 21.56 years with a deviation of 2.20 years. The G^*^Power analysis showed that, with an effect size of 0.25 and a significance level of 0.05 the statistical power of this sample size was 0.96. This met the studys requirements. All 221 college students participated voluntarily. They all signed consent forms.

#### 5.2.2 Experimental procedure

The experimental materials consisted of four historical photographs selected from a pilot study, from which corresponding AI-animated versions were generated using Dreamina AI image animation software. The experiment employed a 2 (Presentation Format: Animated vs. Static) × 2 (Perceived Historical Authenticity: High vs. Low) between-subjects design, with participants randomly assigned to four groups, each comprising approximately 56–57 participants. Experimental procedure: (1) Participants viewed the corresponding vintage photographs (or animated clips), with each image presented for 8 s; (2) Participants completed scales measuring mental imagery, emotional resonance, perceived historical authenticity, emotional state, historical interest, and willingness to share; (3) Participants provided demographic information. The entire experiment lasted approximately 10 min.

#### 5.2.3 Measurement tools

The willingness-to-share scale was adapted from something Berger and Milkman used in 2012. This scale has three points:“I am willing to share these images on social media platforms,” “I would recommend these AI-animated historical photographs to others,” and “If given the opportunity, I would repost or share this type of content” (α = 0.89). The items were rated on a 7-point scale, where 1 represented “not at all” and 7 represented “very much”; higher scores indicated a stronger willingness to share. Participants were then asked to complete the Emotional Resonance Assessment, using the same items as in Experiment 1 (α = 0.92), followed by the Mental Imagery Scale, using the same items as in Experiment 2 (α = 0.92). To ensure the success of the perceived historical authenticity manipulation, this study included a measure of perceived historical authenticity, using the same items as in the pilot study (α = 0.98). The Emotional State and Historical Interest scales used the same items as in Experiments 1 and 2.

### 5.3 Results

#### 5.3.1 Assumption testing

We were prior to conducting the two-way ANCOVA and moderated mediation analyses, assumptions of normality, homogeneity of variance, and homogeneity of regression slopes were examined. No substantial violations were detected. Therefore, the data were considered appropriate for subsequent analyses.

#### 5.3.2 Manipulation check

This experiment used an independent samples *t*-test to analyze whether the perceived historical authenticity manipulation was successful. The results showed that the scores of the high perceived historical authenticity group (M = 5.25, SD = 0.97) were significantly higher than those of the low perceived historical authenticity group (M = 4.84, SD = 1.04), *t*_(219)_ = 3.09, *p* = 0.002, Cohen's *d* = 0.42, indicating a small-to-medium effect, confirming the success of the perceived historical authenticity manipulation. The perceived historical authenticity manipulation, in this experiment was successful because the scores were so different. The perceived historical authenticity really made a difference in this experiment.

#### 5.3.3 Re-examination of the mediating effect

In this experiment we wanted to see how the way old photographs are presented affects people. We used a method called PROCESS Model 4 to do this. We looked at how the presentation format of AI-animated historical photographs impacts emotional resonance. Mental imagery was entered as the mediator, with emotional state and historical interest included as covariates. The results indicate that mental imagery significantly mediates the relationship between presentation format and emotional resonance (β = 0.39, 95% CI [0.28, 0.52]). Specifically, the presentation format of AI-animated historical photographs has a significant positive effect on mental imagery (β = 0.61, SE = 0.08, *p* < 0.001), and mental imagery had a significant positive effect on emotional resonance (β = 0.64, SE = 0.06, *p* < 0.001). Furthermore, after controlling for demographic variables, the mediating role of mental imagery in the relationship between presentation format and emotional resonance remained significant (β = 0.40, 95% CI [0.28, 0.53]). Therefore, this once again supports the mediating role of mental imagery.

#### 5.3.4 Moderation effect analysis

**Figure 2:**
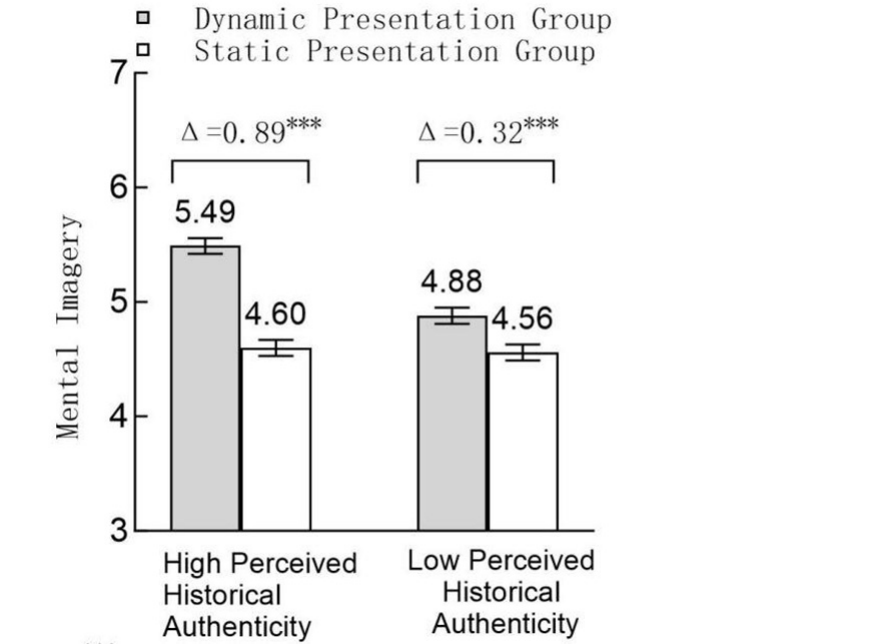
The effects of presentation format and perceived historical authenticity on mental imagery. *** indicates that the mean difference is significant at the 0.001 level.

This experiment employed a two-way ANOVA to examine differences in mental imagery and emotional resonance under different presentation formats of AI-animated historical photographs when participants were exposed to high or low levels of perceived historical authenticity. First, with presentation format and perceived historical authenticity as independent variables, mental imagery as the dependent variable, and emotional state and historical interest as covariates, the results showed a significant main effect of presentation format on mental imagery, *F*_(1, 215)_ = 76.67, *p* < 0.001, ηp^2^ = 0.263, this represents a large effect size according to Cohen's (1988) criteria; the main effect of perceived historical authenticity on mental imagery was also significant, *F*_(1, 215)_ = 21.17, *p* < 0.001, ηp^2^ = 0.09, a medium effect;The interaction effect between the presentation format (dynamic vs. static) and perceived historical authenticity (high vs. low) in AI-animated historical photographs was significant, *F*_(1, 215)_ = 16.18, *p* < 0.001, ηp^2^ = 0.07, a medium effect, supporting the predicted moderation. Further analysis of simple effects (as shown in Figure 2) reveals that when participants reported high perceived historical authenticity, the mental imagery scores in the dynamic presentation group (M = 5.49, SD = 0.49) were significantly higher than those in the static presentation group (M = 4.60, SD = 0.50), *F*_(1, 215)_ = 81.45, *p* < 0.001, ηp^2^ = 0.275, a large effect (mean difference = 0.89);When participants had low perceived historical authenticity, the mental imagery for the dynamic presentation condition (M = 4.88, SD = 0.55) was significantly higher than that for the static presentation condition (M = 4.56, SD = 0.52), *F* = (1, 215) = 10.66, *p* = 0.001, ηp^2^ = 0.047, a small-to-medium effect (mean difference = 0.32; see Figure 2). The substantially larger effect size under high authenticity conditions supports the predicted moderating role of perceived historical authenticity. So the results support Hypothesis 3.

**Figure 3:**
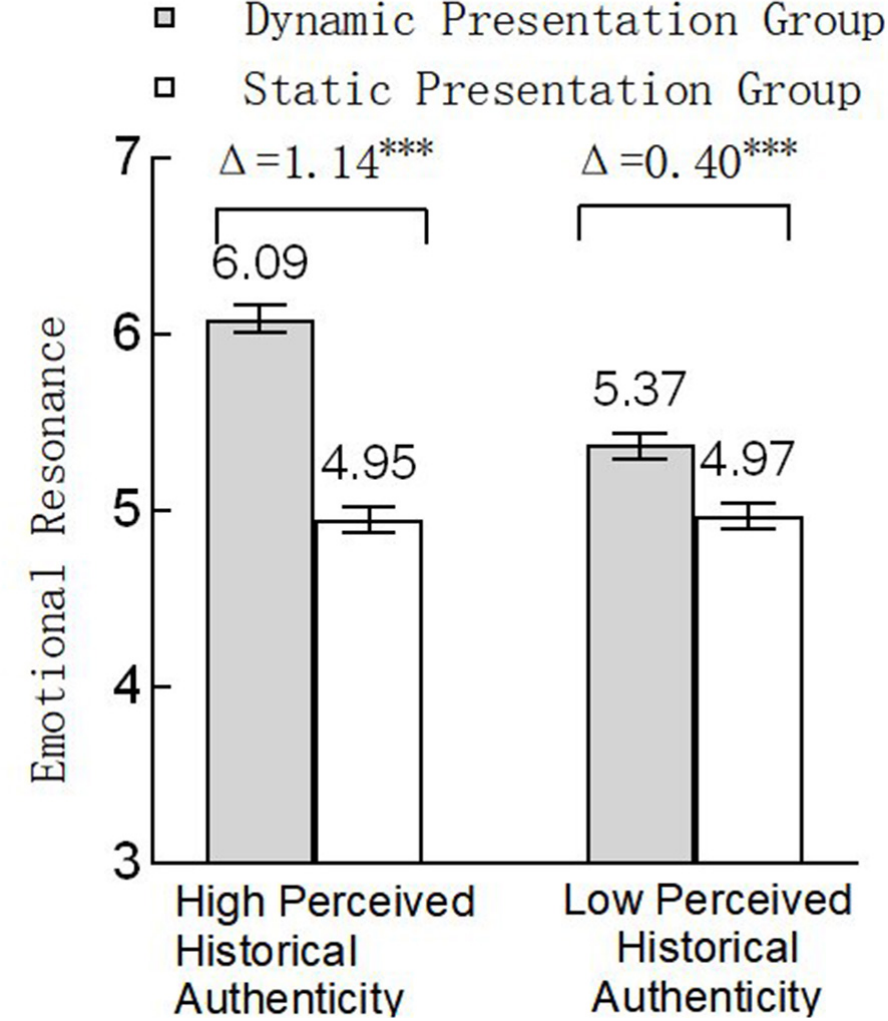
The effects of presentation format and perceived historical authenticity on emotional resonance. *** indicates that the mean difference is significant at the 0.001 level.

Next a study was done to see how presentation format and historical accuracy affect how people feel. We are analyzing with presentation format and perceived historical authenticity as independent variables, emotional resonance as the dependent variable, and emotional state and historical interest as covariates. The results showed that presentation format had a significant main effect on emotional resonance, *F*_(1, 215)_ = 108.97, *p* < 0.001, ηp^2^ = 0.336, it indicates a large effect size, suggesting substantial practical significance;the main effect of perceived historical authenticity on emotional resonance was also significant, *F*_(1, 215)_ = 22.06, *p* < 0.001, ηp^2^ = 0.093; the interaction effect between the presentation format of AI-animated historical photographs (dynamic vs. static) and perceived historical authenticity (high vs. low) was significant, *F*_(1, 215)_ = 24.84, *p* < 0.001, ηp^2^ = 0.104. Further analysis of simple effects (as shown in Figure 3) reveals that when participants experienced high perceived historical authenticity, the mental imagery scores in the dynamic presentation group (M = 6.09, SD = 0.59) were significantly higher than those in the static presentation group (M = 4.95, SD= 0.55), *F* = (1,215) = 118.683, *p* < 0.001, ηp^2^ = 0.356;When participants had low perceived historical authenticity, the mental imagery from the dynamic presentation (M = 5.37, SD = 0.59) was significantly higher than that from the static presentation group (M = 4.97, SD = 0.46), *F* = (1,215) = 14.12, *p* < 0.001, ηp^2^ = 0.062. The above results test Hypothesis 4. Furthermore, after controlling for demographic variables, the interaction effect between the different presentation formats of AI-animated historical photographs and participants' varying levels of perceived historical authenticity on mental imagery remains significant (*F* = (1, 213) = 16.15, *p* < 0.001, ηp^2^ = 0.07), and the interaction effect between these two factors and emotional resonance (*F* = (1, 213) = 25.48, *p* < 0.001, ηp^2^ = 0.107).

#### 5.3.5 Moderated mediation effects

In this experiment, the moderated mediation effect was tested using PROCESS Model 8. The presentation format of AI-animated historical photographs (static/dynamic) served as the independent variable, emotional resonance as the dependent variable, mental imagery as the mediating variable, and historical authenticity (high/low) as the moderating variable, with emotional state and historical interest as covariates. The results showed that, with mental imagery as the mediating variable, the interaction effect between the presentation format of AI-animated historical photographs (static/dynamic) and perceived historical authenticity (high/low) on emotional resonance was significant (β = 0.44, 95% CI [0.17, 0.70]), because the confidence interval did not include zero, the moderated mediation effect was considered statistically significant. So it confirms Hypothesis 5. Specifically, when the perceived historical authenticity was high, the mediating effect of mental imagery was significant (β = 0.49, 95% CI [0.34, 0.66]); when the perceived historical authenticity was low, the mediating effect of mental imagery was also significant (β = 0.17, 95% CI [0.07, 0.31]). Furthermore, after controlling for demographic variables, mental imagery continued to play a significant mediating role in the interaction between presentation format and perceived historical authenticity on emotional resonance (β = 0.45, 95% CI [0.18, 0.71]).

#### 5.3.6 Analysis of the chain mediation effect of mental imagery and emotional resonance

**Figure 4:**
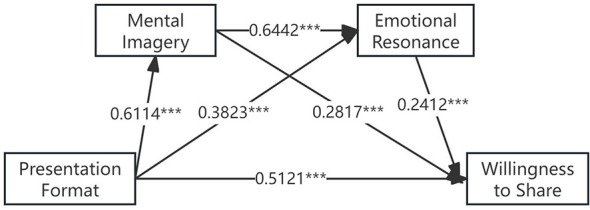
Path coefficients of variables in the serial mediation model. ***indicates that the mean difference is significant at the 0.001 level.

We used linear regression analysis to examine the effect of emotional resonance on the willingness to share. The results revealed that emotional resonance has a significant positive effect on the willingness to share (β = 0.587, *t*_(219)_ = 10.72, *p* < 0.001), confirming that emotional resonance significantly and positively influences the willingness to share. To further analyze how presentation format influences willingness to share through mental imagery and emotional resonance, this study tested the chain mediation model using Process Model 6. Presentation format served as the independent variable, willingness to share as the dependent variable, mental imagery and emotional resonance as mediating variables, and emotional state and historical interest as control variables. The results indicate that the chained mediation effect of presentation format on willingness to share via mental imagery and emotional resonance is significant (β = 0.095, 95% CI [0.05, 0.15]). The confidence interval excluded zero, indicating that the chained mediation effect was statistically significant. The path coefficients among the variables are shown in Figure 4, supporting Hypothesis 6.

### 5.4 Theoretical interpretation and model integration

The results of Experiment 3 indicate that the emotional effects of dynamic visual cues are not universally stable but are significantly moderated by perceived historical authenticity. Specifically, when viewers perceive the imagery to have high perceived historical authenticity, they are more likely to incorporate it into their existing historical cognitive framework, thereby enhancing mental imagery generation and further strengthening emotional resonance; when perceived historical authenticity is low, individuals are more likely to maintain cognitive distance, limiting internal mental simulation and emotional resonance. This finding reveals that authenticity does not merely influence emotional intensity but acts at the stage where visual stimuli are transformed into mental imagery, thereby constituting a critical boundary condition for the emotional effects of AI-animated historical photographs. Furthermore, the study found that mental imagery and emotional resonance form a chain-like pathway in the process by which presentation formats influence sharing behavior, indicating that emotional experience is not only the result of perceptual processing but also a key psychological mechanism driving information dissemination.

Synthesizing the experimental results, this study constructs and validates a theoretical model that integrates visual presentation characteristics, psychological processing mechanisms, and communication behavior, revealing how AI-animated historical photographs transform from perceptual stimuli into emotional experiences and further influence social communication.

## 6 Discussion

### 6.1 Key findings

This study investigated how AI-animated historical photographs influence viewers' emotional responses and communication intentions through a series of three experiments. The findings suggest that the effect of dynamic presentation on emotional resonance is not direct; rather, it operates through underlying psychological processes involving mental imagery and authenticity perception.

First, dynamic presentation elicited significantly higher levels of emotional resonance than static presentation. This finding is consistent with previous research demonstrating that motion cues enhance emotional engagement and psychological immersion in visual media (Zhang Z. et al., 2024). However, the present findings extend this literature in an important way. Previous studies have primarily examined motion effects in commercial advertising, entertainment media, and digital storytelling contexts, where emotional engagement is often driven by attention capture and narrative immersion. In contrast, the current study demonstrates that similar effects emerge in historical communication settings, where audiences are not merely processing visual stimuli but are also evaluating representations of the past. This suggests that the emotional consequences of motion cues may extend beyond entertainment contexts and play a meaningful role in historical interpretation and cultural memory construction (Giaccardi, 2012; Champion, 2016). This is not just because dynamic presentation is more visually appealing or grabs attention. Motion cues appear to facilitate viewers' mental simulation of historical events, allowing them to experience historical scenes as psychologically vivid and personally meaningful experiences. So the emotional advantage of presentation is better understood as a process of psychological simulation.

Second, the results identify mental imagery as the primary psychological mechanism underlying the effectiveness of dynamic presentation. The experiments showed that dynamic presentation increased resonance mainly by helping viewers imagine historical situations. This finding aligns with research of Miskolczi (2025). Emotional state and historical interest did not explain the effect. These findings suggest that audience engagement with AI-animated historical photographs depends primarily on the extent to which viewers can construct vivid mental representations of the depicted historical context, not their current mood or historical interest.

Third, perceived historical authenticity significantly shaped the effectiveness of dynamic presentation. When viewers think the images are historically credible dynamic presentation works better. When authenticity perceptions were low, dynamic cues were more likely to be interpreted as artificial manipulations, thereby weakening emotional engagement. This supports earlier evidence of Huang's research (Huang et al., 2025). This shows that people judge AI-animated historical photographs not only by their visual appearance but also by whether they believe them to be historical.

Finally, dynamic presentation influenced not only emotional responses but also subsequent communication behavior. Dynamic presentation makes people more willing to share the images. This happens because stronger mental images lead to emotions, which in turn make people want to share. Overall the findings show that restored historical images have different effects. Dynamic presentation is not always more effective than presentation. Its influence depends on whether motion cues help people imagine whether people believe the images are authentic and whether the resulting emotions are strong enough to motivate sharing. This is in line with the research of Park. Park show that emotionally engaging AI-animated historical photographs can increase users' willingness to interact with and share content online (Park et al., 2025).

The study also shows that people's emotions and sharing intentions are influenced by AI-animated historical photographs. The effect of presentation, on people's emotions and communication behavior is significant.

### 6.2 Theoretical contributions

This study makes three theoretical contributions.

First, this study extends the literature on dynamic visual communication by identifying mental imagery as a key mechanism through which AI-animated historical photographs influence emotional responses. More importantly, the findings help differentiate Mental Imagery Theory from broader engagement-oriented frameworks such as Media Richness Theory and Transportation Theory. While these perspectives explain why dynamic media may attract attention and increase immersion, the present findings demonstrate that emotional resonance is ultimately facilitated through the formation of internal mental simulations. This provides empirical evidence supporting the unique explanatory value of Mental Imagery Theory in the context of AI-animated historical photographs. Previous studies said dynamic images work well because they grab our attention are vivid or stand out. Rather than merely increasing perceptual vividness, dynamic historical imagery appears to facilitate simulation-based cognitive processing, thereby strengthening emotional resonance (Finke, 1985; MacInnis and Price, 1987). In short they work through simulation-based feelings not just being vivid. By showing that motion cues make emotions stronger through imagery this study changes how we explain things from outside stimulation to inside our minds.

Second, this study contributes to authenticity research by demonstrating that perceived historical authenticity functions as a psychological boundary condition in audience responses to AI-animated historical photographs. Previous authenticity studies have largely focused on museums, heritage tourism, and historical exhibitions (Waitt, 2000; Chhabra et al., 2003), emphasizing the importance of credibility and trust. The present findings extend this perspective by showing that authenticity perceptions also influence whether technologically generated visual cues are cognitively accepted and emotionally integrated. Thus, authenticity operates not only as an evaluative judgment but also as a mechanism that shapes emotional processing in AI-mediated historical communication.

Third, this study helps us understand communication better. It shows how what we see turns into sharing. The results say that dynamic presentation makes us imagine more imagining makes emotions stronger and stronger emotions make us want to share. So emotional resonance helps turn what we see into sharing. This means AI-animated historical photographs isn't just new and cool. It can turn what we see into something that means something to us emotionally.

In all this study gives us a framework that connects how things are presented what we imagine how real we think it is and if we want to share. It gives an explanation of how AI-animated historical photographs are processed emotionally and shared socially. At the same time, the findings should be interpreted within the demographic and cultural context in which the study was conducted, highlighting the importance of future validation across broader populations and cultural settings.

### 6.3 Implications for AI and digital culture research

From the broader context of digital culture, this study offers several noteworthy insights.

First, the emotional impact of AI-animated historical photographs is not entirely determined by technical precision but is closely related to its ability to trigger viewers' contextual imagination. The findings suggest that the emotional impact of AI-animated historical photographs depends less on technical sophistication and more on its capacity to facilitate contextual imagination. Accordingly, designers of AI-enhanced historical content should prioritize the provision of narrative and contextual cues that enable audiences to mentally reconstruct historical situations. Such cues may contribute more substantially to emotional engagement than visual enhancement alone.

Second, the role of perceived historical authenticity in AI content dissemination warrants further attention. Overemphasizing technical processing may weaken emotional resonance, whereas retaining a certain historical texture and traces of time can actually help strengthen emotional connections. This suggests that AI content design does not necessarily need to aim for “complete realism,” but rather requires striking a balance between technical expression and a sense of history.

Third, the findings of this study indicate that emotional resonance is a key driver of AI visual content dissemination. Sharing behavior is often not a rational decision but rather an extension of emotion. When viewers form emotional experiences during the viewing process, they are more likely to perpetuate them through forwarding or discussion.

### 6.4 Ethical implications of AI-animated historical photographs

The increasing use of AI technologies to animate historical photographs raises important ethical considerations. While AI animation may enhance emotional engagement and historical accessibility, it also introduces the possibility of altering public perceptions of historical reality (Gendron and Barrett, 2018). Unlike traditional restoration techniques that primarily improve image quality, AI animation adds behavioral and temporal information that did not originally exist in the historical record (Jones, 2017).

As the present findings indicate, audiences often respond emotionally to animated historical photographs when they perceive them as authentic. Although this emotional engagement may enhance historical communication, it also creates the risk that viewers may interpret algorithmically generated movements as historically accurate representations (Sundar, 2020). Such interpretations may unintentionally blur the boundary between historical documentation and technological reconstruction.

Therefore, designers, museums, educational institutions, and digital heritage practitioners should carefully consider issues of transparency and disclosure when presenting AI-animated historical photographs (Floridi et al., 2018). Clearly communicating the reconstructed nature of AI-animated motion may help preserve historical integrity while still allowing audiences to benefit from the emotional and educational value of these technologies. Future research should further examine how different disclosure strategies influence audience trust, authenticity perceptions, and historical understanding.

### 6.5 Research limitations and future research agenda

It should be noted that this study has several limitations.

First, the sample consisted primarily of university students. Given potential age-related and cultural differences in media use and historical engagement, caution should be exercised when generalizing the findings to broader populations. In the future, the studies should recruit more diverse samples to enhance the external validity of the findings. In addition, all participants were recruited from a single cultural context. Perceptions of historical authenticity, emotional resonance, and attitudes toward AI-animated media may vary across cultural backgrounds (Hofstede, 2001). Therefore, the generalizability of the present findings to other cultural settings remains uncertain. Future studies should conduct cross-cultural comparisons to examine whether the observed psychological mechanisms remain stable across different historical traditions and media environments.

Second, the study looks at photographs of people. Other kinds of historical images like videos of wars or family videos may affect people in different ways. Historical images, like these may make people feel differently. The study does not look at these kinds of images like local cultural materials. Future research could further compare the differences in emotional pathways across various visual materials.

Third, this study treated “dynamic presentation” as a single variable, but different generation methods (such as micro-expression animations, deep-learning-generated videos, and 3D reconstructions) may produce distinct experiences. A detailed analysis of these technical differences would aid in a more accurate understanding of AI visual effects.

Finally, the future studies could incorporate objective physiological measures, such as eye-tracking, psychophysiological indicators, or neuroimaging techniques, to provide a more comprehensive understanding of the cognitive and affective processes underlying responses to AI-animated historical photographs. It might give us clear evidence. We could study how people look at visuals how their bodies react or how their brains work. This can help us understand emotions better.

## Data Availability

The original contributions presented in the study are included in the article/supplementary material, further inquiries can be directed to the corresponding author.
